# Fruit Bromelain-Derived Peptide Potentially Restrains the Attachment of SARS-CoV-2 Variants to hACE2: A Pharmacoinformatics Approach

**DOI:** 10.3390/molecules27010260

**Published:** 2022-01-01

**Authors:** Trina Ekawati Tallei, Fatimawali, Ahmad Akroman Adam, Mona M. Elseehy, Ahmed M. El-Shehawi, Eman A. Mahmoud, Adinda Dwi Tania, Nurdjannah Jane Niode, Diah Kusumawaty, Souvia Rahimah, Yunus Effendi, Rinaldi Idroes, Ismail Celik, Md. Jamal Hossain, Talha Bin Emran

**Affiliations:** 1 Department of Biology, Faculty of Mathematics and Natural Sciences, Sam Ratulangi University, Manado 95115, Indonesia; 2 The University Centre of Excellence for Biotechnology and Conservation of Wallacea, Institute for Research and Community Services, Sam Ratulangi University, Manado 95115, Indonesia; fatimawali@unsrat.ac.id (F.); niodejane@unsrat.ac.id (N.J.N.); 3 Pharmacy Study Program, Faculty of Mathematics and Natural Sciences, Sam Ratulangi University, Manado 95115, Indonesia; 17101105001@student.unsrat.ac.id; 4 Dentistry Study Program, Faculty of Medicine, Sam Ratulangi University, Manado 95115, Indonesia; ahmad_adam@ymail.com; 5 Department of Genetics, Faculty of Agriculture, University of Alexandria, Alexandria 21545, Egypt; monaahmedma@yahoo.com; 6 Department of Biotechnology, College of Science, Taif University, P.O. Box 11099, Taif 21944, Saudi Arabia; elshehawi@hotmail.com; 7 Department of Food Industries, Faculty of Agriculture, Damietta University, Damietta 34511, Egypt; emanmail2005@yahoo.com; 8 Department of Dermatology and Venereology, Faculty of Medicine, University of Sam Ratulangi, RD Kandou Hospital, Manado 95163, Indonesia; 9 Department of Biology, Faculty of Mathematics and Natural Sciences Education, Universitas Pendidikan Indonesia, Bandung 40154, Indonesia; diah.kusumawaty@upi.edu; 10 Food Technology Study Program, Department of Food Industrial Technology, Faculty of Agroindustrial Technology, Universitas Padjadjaran, Jatinangor 45363, Indonesia; souvia@unpad.ac.id; 11 Department of Biology, Faculty of Science and Technology, Al-Azhar Indonesia University, Jakarta 12110, Indonesia; effendiy@uai.ac.id; 12 Department of Pharmacy, Faculty of Mathematics and Natural Sciences, Universitas Syiah Kuala, Kopelma Darussalam, Banda Aceh 23111, Indonesia; rinaldi.idroes@unsyiah.ac.id; 13 Department of Pharmaceutical Chemistry, Faculty of Pharmacy, Erciyes University, Kayseri 38039, Turkey; ismailcelik@erciyes.edu.tr; 14 Department of Pharmacy, State University of Bangladesh, 77 Satmasjid Road, Dhanmondi, Dhaka 1205, Bangladesh; jamal.du.p48@gmail.com; 15 Department of Pharmacy, BGC Trust University Bangladesh, Chittagong 4381, Bangladesh

**Keywords:** bromelain, peptide, SARS-CoV-2 variants, in silico, receptor-binding domain, RBD mutation, COVID-19, molecular dynamics simulation, peptide-protein interaction

## Abstract

Before entering the cell, the SARS-CoV-2 spike glycoprotein receptor-binding domain (RBD) binds to the human angiotensin-converting enzyme 2 (hACE2) receptor. Hence, this RBD is a critical target for the development of antiviral agents. Recent studies have discovered that SARS-CoV-2 variants with mutations in the RBD have spread globally. The purpose of this in silico study was to determine the potential of a fruit bromelain-derived peptide. DYGAVNEVK. to inhibit the entry of various SARS-CoV-2 variants into human cells by targeting the hACE binding site within the RBD. Molecular docking analysis revealed that DYGAVNEVK interacts with several critical RBD binding residues responsible for the adhesion of the RBD to hACE2. Moreover, 100 ns MD simulations revealed stable interactions between DYGAVNEVK and RBD variants derived from the trajectory of root-mean-square deviation (RMSD), radius of gyration (Rg), and root-mean-square fluctuation (RMSF) analysis, as well as free binding energy calculations. Overall, our computational results indicate that DYGAVNEVK warrants further investigation as a candidate for preventing SARS-CoV-2 due to its interaction with the RBD of SARS-CoV-2 variants.

## 1. Introduction

As of 21 October 2021, the WHO (World Health Organization) had received reports of 241,886,635 confirmed coronavirus disease 2019 (COVID-19) cases worldwide, with 4,919,755 deaths. Although global vaccination campaigns are currently underway, it remains unclear how long the vaccine will provide immune defense against infection or if currently approved vaccinations will be sufficient to protect against emerging virus variants. Currently, numerous SARS-CoV-2 variants are emerging worldwide. The Centers for Disease Control and Prevention (CDC) have classified the SARS-CoV-2 variants as variants of interest (VOI), variants of concern (VOC), and variants of high consequence (VOHC). Additionally, the WHO has given Greek letters to SARS-CoV-2 variants.

Three variants that have risen to prominence in their respective countries and have sparked concern are the B.1.1.7, B.1.351, and P.1 lineages [1]. The B.1.1.7 lineage (alpha variant) was first described in the United Kingdom, while the B.1.351 lineage (beta variant) was initially reported in South Africa, and the P.1 lineage (gamma variant) was first reported in Brazil. Each of these three variants contains the N501Y mutation, which converts the amino acid asparagine (N) to tyrosine (Y) in the receptor-binding domain (RBD) subunit S1b of the glycoprotein spike. The B.1.1.7 lineage only has the N501Y mutation, while the B.1.351 and P.1 lineages have multiple mutations in the spike protein, including K417N, E484K, and N501Y [1,2,3,4,5]. Notably, SARS-CoV-2 mutations in the RBD were not confined to these three variants. To date, several variants with various mutations in different positions have circulated world-wide, including the California, New York, Scotland, Nigeria, and Indian variants [6,7,8,9,10].

To trigger cell entry and infection, the SARS-CoV-2 spike protein interacts directly with an enzyme known as the human angiotensin-converting enzyme 2 (hACE2) receptor. What is more concerning is that some of these variants have been shown to bind to the hACE2 receptor more effectively [2]. While hACE2 receptors are located on the surfaces of cells in a variety of tissues, they are particularly prevalent in the lungs [3,4]. Concerns have arisen since it is suspected that some of the COVID-19 vaccines currently in use are less effective against these variants [5,6,7]. As a result, researchers are also seeking COVID-19 antidotes.

Recently, several studies have been directed toward investigating a peptide (a small part of a protein) that can prevent the binding of the SARS-CoV-2 RBD to hACE2 [8,9,10]. For example, an antiviral peptide is a form of antiviral agent that is intended to be used as a therapeutic agent against a particular disease, for example, COVID-19. According to a report, 124 studies involving peptides were conducted in the search for an antidote for COVID-19. Of these, there were several clinical trials in the management of COVID-19, including immunomodulatory (5 trials), homeostasis regaining (8 trials), vaccination (9 trials), and antiviral activity trials (4 trials) [11].

Since peptides are more effective and precise than small-molecule drugs, they may be better tolerated [12]. Moreover, unlike other antiviral drugs, peptides have no toxicity to human cells [13,14]. However, despite their high therapeutic potential, some peptides have failed to reach clinical trials because of their toxicity (hemolytic activity) [15]. Peptide synthesis can also be easily implemented and tuned. On the other hand, small molecules often necessitate the creation of new time-consuming and expensive synthetic techniques [16]. Antiviral peptides outperform traditional antiviral drugs [17] because they are more effective, have a smaller molecular weight, and have fewer side effects [18]. To date, the FDA has approved more than 60 peptide-based drugs. However, more than 150 peptides are still undergoing advanced clinical trials [19]. Enfuvirtide is an HIV-1 fusion inhibitor linear synthetic peptide with a length of 36-amino acids and is an FDA-approved antiviral peptide [20]. Thus, peptides are molecules that can be tested against SARS-CoV-2 to potentially develop new drugs to treat COVID-19 [21].

Bioactive peptides are defined as peptide sequences contained within a protein that have a beneficial effect on bodily functions and/or have a beneficial effect on human health in addition to the protein’s known nutritional value. They typically have a length of 3–20 amino acid residues [22]. Bioactive peptides can be obtained from a variety of sources, including animals, plants, and microorganisms. They can also be derived from a variety of proteins. When protein is consumed, it is digested in the digestive tract, resulting in the formation of peptides with numerous beneficial properties for the body. One of the proteins that has been widely researched and is known to have many health benefits is bromelain. It is a mixture of various cysteine proteinases with similar amino acid sequences and is found in pineapple fruits and stems [1,23,24,25]. Bromelain has been shown to reduce the expression of ACE-2 and TMPRSS2 in VeroE6 cells, as well as to significantly reduce the expression of the S-Ectodomain of SARS-CoV-2 [26]. According to our previous in silico research, bromelain has a high binding affinity for various RBD variants and binds directly at the binding site between RBDs and hACE2. This suggests that bromelain has the potential to inhibit SARS-CoV-2 attachment to hACE2 [27]. In one study, the bromelain peptide biomarker DYGAVNEVK was found in the plasma of bromelain-treated mice. This peptide is one of bromelain’s four proteolytically active proteins, which contribute to its therapeutic properties [28]. In the present study, we performed molecular docking analysis and an MD simulation study of the peptide DYGAVNEVK against several variants of the SARS-CoV-2 RBD.

## 2. Materials and Methods

### 2.1. Multiple Sequence Alignment of Wild-Type RBD and Its Variants 

Multiple sequence alignment (MSA) techniques are a collection of algorithmic solutions for aligning evolutionarily related sequences. MSA of the amino acid sequences of the wild-type (WT) receptor-binding domain (RBD) and its variants was performed using the UCSF Chimera package (release 1.15) [29].

### 2.2. Three-Dimensional Structures of the Bromelain-Derived Peptide and the RBD Variants

The three-dimensional (3D) structures of the bromelain-derived peptide DYGAVNEVK and RBD variants were modeled and minimized using the SWISS-MODEL web server (https://swissmodel.expasy.org/interactive; accessed on 9 September 2021) [30,31]. Moreover, the two-dimensional (2D) structure was generated in PDBsum (http://www.ebi.ac.uk/thornton-srv/databases/pdbsum; accessed on 10 September 2021) [32]. The WT RBD was retrieved from a protein data bank with PDB ID 6M0J (https://www.rcsb.org/structure/6M0J; accessed on 12 September 2021). The structure control of the variants’ model structures was checked by MolProbity Structure Assessment and a Ramachandran plot.

### 2.3. Physicochemical Properties Analysis

Calculation of the theoretical physicochemical properties of the peptide was performed using PepDraw (http://www.tulane.edu/~biochem/WW/PepDraw/; accessed on 14 September 2021) and Protparam (https://web.expasy.org/cgi-bin/protparam/protparam; accessed on 14 September 2021) [33].

### 2.4. Allergenicity and Toxicity Prediction

The theoretical allergenicity of the peptide was calculated using the Allergen FP v.1.0 webserver [34]. The toxicity prediction was conducted using ProTox-II (https://tox-new.charite.de/protox_II/; accessed on 14 September 2021) [35].

### 2.5. IC_50_ Prediction

The half-maximal inhibitory concentration (IC_50_) of the peptide’s antiviral activity was predicted using the AVP-IC 50Pred server (http://crdd.osdd.net/servers/ic50avp/; accessed on 15 September 2021) [36] by selecting RSV/INFV/HSV prediction model 23 and the default parameters. The results in the output were divided into 4 groups based on the predicted IC_50_ values: (1) <1 μM: highly effective; (2) 1–10 μM: effective; (3) 11–100 μM: moderately effective; (4) >100 μM: least effective.

### 2.6. Molecular Docking

The blind molecular docking assay between the bromelain-derived peptide and RBDs was performed using the HADDOCK2.2 Web Server (https://milou.science.uu.nl/services/HADDOCK2.2/; accessed on 16 September 2021) [37]. Chimera software version 1.15 [29] was used to predict the peptide position between RBD and hACE2.

### 2.7. Equilibrium Dissociation Constant Analysis

The equilibrium dissociation constant (K_D_) is defined by the ratio of ligand receptor unbinding to binding rates. For each complex, the K_D_ was predicted by PRODIGY (PROtein binDIng enerGY) (https://bianca.science.uu.nl/prodigy/; accessed on 16 September 2021) [38]. 

### 2.8. Analysis of MM-GBSA Free Energy

Using the molecular mechanics-generalized born surface area (MM-GBSA) approach, the binding-free energy (BFE) of the protein-peptide complex was calculated as the difference between the energy of the bound complex and that of the unbound protein and peptide. The calculations were performed using the HawkDock web server (http://cadd.zju.edu.cn/hawkdock/; accessed on 17 September 2021) [39]. 

### 2.9. Analysis of the Complex Interface

The general content of the interface area resulting from molecular docking tests was analyzed using the PDBsum database for the structural analysis of 3D structures (EMBL-EBI; http://www.ebi.ac.uk/thornton-srv/databases/pdbsum/Generate.html; accessed on 18 September 2021). The 2D visualization of this interaction was generated using LigPlot+ [40].

### 2.10. Molecular Dynamics Simulations Study

MD simulations were performed using Gromacs version 2019.2 via WebGRO for macromolecular simulations (https://simlab.uams.edu/; accessed on 21 July 2021) [41]. The topology of the bromalin peptide and RBD protein complex was established by choosing the am-ber99sb-ildn [42] force field and the simple point-charge (SPC) water model. For the MD system, the triclinic water box was preferred and neutralized by adding the appropriate 0.15 M NaCl salt. The energy minimization of the created system was carried out in 5000 steps with the steepest descent integrator. The canonical ensemble NVT (moles (N), volume (V), and temperature (T)) equilibrium phase of the system was carried out at 300 K using the 0.3 ns duration V-rescale method [43], and the isothermal-isobaric ensemble NPT (moles (N), pressure (P), and temperature (T)) equilibrium phase was carried out using the Parrinello–Rahman method [44] at 0.3 ns under 1 atm of pressure. The MD simulation was performed using a leapfrog MD integrator to form 5000 frames with a duration of 100 ns. Root-mean-square deviation (RMSD), root-mean-square fluctuation (RMSF), and radius of gyration (Rg) trajectory analyses were performed.

### 2.11. Binding-Free Energies Calculation

The molecular mechanics Poisson–Boltzmann surface area (MM-PBSA) BFE calculation is widely used to analyze the stability and bonding strength of protein-ligand, protein-peptide, and protein-protein complexes [45]. The BFE consists of polar solvation energies, solvent accessible surface area (SASA) energy, and electrostatic and van der Waals interactions. In this study, binding free energy calculations for the RBD S1b units and bromelain peptide complexes of SARS-CoV-2 variants were performed using the MM-PBSA method with 60 frames between 40 and 70 ns from the MD trajectory. The ‘MmPbSaStat python’ script provided in g_mmpbsa was used for the average binding energy calculations [45,46].

## 3. Results and Discussion

The spread of SARS-CoV-2 variants across several continents is a significant source of concern for global human health. The variants are rapidly transmissible and quickly become prevalent in populations. Notably, the spike (S) protein has accumulated a large number of mutations, particularly within the amino-terminal domain (NTD) and the RBD. The emergence of mutations in this spike has direct implications for the high rate of viral infection since a conformational change in the RBD has resulted in stronger binding to the ACE2 receptor. Single amino acid substitutions should be monitored because they can have phenotypic consequences [47,48].

**Figure 1:**
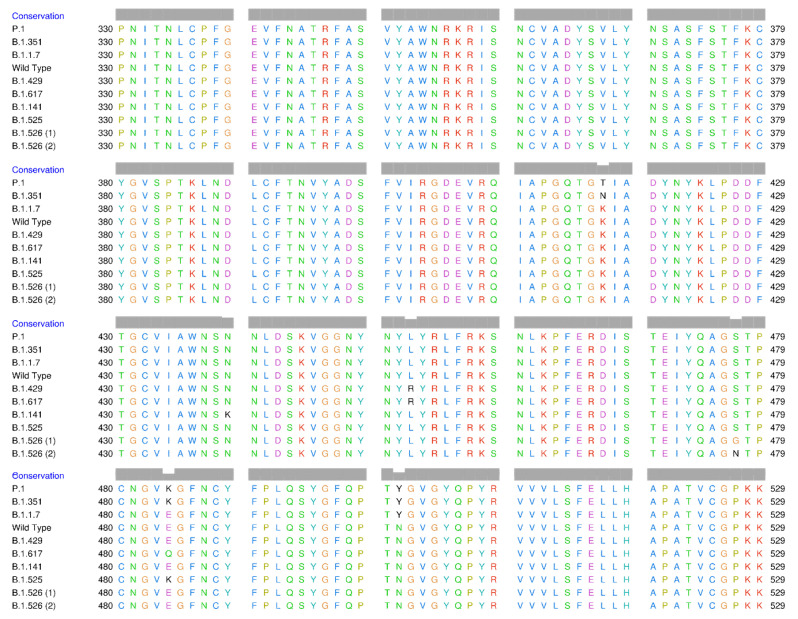
The genomic structure of the S glycoprotein of SARS-CoV-2 showing the position of each gene and the impactful mutations in the RBD.

The MSA results for the RBD wild type and its variants are presented in Figure 1 and Table 1. These results show the positions of mutations in the RBD of SARS-CoV-2. The following mutations were found in the RBD and are listed in Table 1: K417N (lysine, positive polar to asparagine, neutral polar); K417T (lysine, neutral polar to threonine, neutral polar); N439K (asparagine, neutral polar to lysine, positive polar); L452R (leucine, neutral nonpolar to arginine, positive polar); S477G (serine, neutral polar to glycine, neutral nonpolar); S477N (serine, neutral polar to asparagine, neutral polar), E484K (glutamate, negative polar to lysine, positive polar); E484Q (glutamate, negative polar to glutamine, neutral polar); and N501Y (asparagine, neutral polar to tyrosine, neutral polar). According to this explanation, since the K417T, S477N, and N501Y mutations do not change the charge or polarity of the amino acid residues, they hypothetically do not cause a conformational change in the RBD. However, since other mutations undergo changes in the charge and polarity of the amino acid residues, these are likely to change the conformation and have an impact on the attachment of the RBD to hACE2. If the mutation includes negatively charged, polar, and hydrophilic amino acids, there will be an increase in RBD stability [49].

### 3.1. The 3D and 2D Structures of Bromelain-Derived Peptide

**Figure 2:**
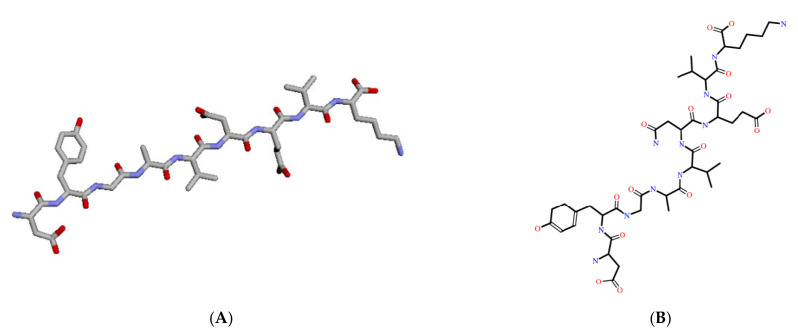
The 3D (**A**) and 2D (**B**) structures of bromelain-derived peptide ASP-TYR-GLY-ALA-VAL-ASN-GLU-VAL-LYS (DYGAVNEVK).

The 3D and 2D structures of the bromelain-derived peptide are presented in Figure 2. The structure was generated from the sequence DYGAVNEVK (ASP-TYR-GLY-ALA-VAL-ASN-GLU-VAL-LYS). The 3D structure was used for peptide-protein docking analysis. The position of the sequence was demonstrated in the previous study [28].

### 3.2. Physicochemical Properties

The peptide has a molecular mass of 993.4751 Da. Ideally, small-molecule drugs with typical molecular weights of 500 Da or less are preferred for oral bioavailability [50]. However, if the peptide is between 500 and 1000 Da, there is still hope for further exploration, as it represents an enormous opportunity for those willing to explore new frontiers of drug discovery [51].

The peptide has an isoelectric point of 4. This means at a pH 4, the peptide carries no net electrical charge (that is, it is electrically neutral) based on the statistical mean. The overall net charge of the peptide at pH 7 is −1. The hydrophobicity scale for the peptide is 18.84 kcal/mol based on the free energy of transfer. The more positive the hydrophobicity scale, the more hydrophobic the molecule. Peptides containing a high proportion of hydrophobic amino acids will demonstrate a detrimental effect on their solubility in water. When designing soluble peptides, a good rule of thumb is to charge 1 out of every 5 amino acids. If this cannot be accomplished, non-essential amino acids in the peptide sequence can be replaced with charged residues [52]. This, of course, has the potential to alter the peptide’s nature [53]. Consequently, substitutions should be carefully considered. 

The amino acid composition and the number of peptide bonds present in a peptide are used to calculate the predictability of its molar extinction coefficient. The predicted molar extinction coefficient of the peptide is 1490 M^−1^ cm^−1^. The N-terminal of the sequence considered is D (Asp). Based on this, the estimated half-life is 1.1 h in mammalian reticulocytes in an in vitro study. The instability index (II) is computed to be −13.18; therefore, this classifies the peptide as stable. Additionally, the peptide is water soluble. Good oral bioavailability will occur if a drug candidate exhibits optimal permeability and solubility at the absorption site [54].

### 3.3. Allergenicity and Toxicity Prediction

Allergic reactions are difficult to predict because they entail complicated interactions between a chemical (allergen) and the immune system [55]. The allergen initiates a Th2 response, which causes B cells to generate IgE and activates eosinophils [56]. Eosinophil accumulation in tissues can be extremely detrimental, as it results in inflammation and tissue damage [57]. According to the prediction, the studied peptide has the potential to be an allergen with a Tanimoto similarity index of 0.71. The higher the value, the closer the two structures are [34]. Additionally, the prediction indicates that the propensity for in vitro aggregation is 0.00.

The toxicity analysis considered several parameters, including the LD_50_, predicted toxicity class, hepatotoxicity, carcinogenicity, immunotoxicity, mutagenicity, and cytotoxicity. Toxicity levels are classified as follows: classes 1 and 2 (fatal if swallowed), class 3 (toxic if swallowed), class 4 (harmful if swallowed), class 5 (possibly harmful if swallowed), and class 6 (nontoxic) [58]. The predicated LD_50_ of the peptide is 500 mg/kg, which classified the molecule in class 4, indicating it is harmful if swallowed. It is predicted to be non-hepatotoxic with a probability of 0.93. Additionally, it is not shown to be carcinogenic, immunotoxic, mutagenic, and cytotoxic, with probabilities of 0.64, 0.99, 0.79, and 0.69, respectively.

### 3.4. The Predicted IC_50_ Value of Bromelain-Derived Peptide

The predicted IC50 value of the bromelain-derived peptide using the AVP-IC 50Pred server IC_50_ was 40.67 μM (moderately effective, predicted by support vector machines (SVMs)) and 6.85 μM (effective, predicted by random forest (RF)). The IC_50_ value is a quantitative measure of the amount of a molecule or drug required to inhibit up to half (50%) of a specific biological process. Since these values are only estimations, further in vitro research is required to validate them. In comparison, the AHB1 and AHB2 peptides have been shown to neutralize SARS-CoV-2 with IC_50_ values of 35 and 16 nM, respectively [16]. The peptides P9R and PR had IC_50_ values of 0.9 μg/mL and 2.4 μg/mL against SARS-CoV-2, respectively [59]. The amino acid composition of peptides may affect their inhibitory activity [60,61].

### 3.5. Analysis of the Interaction between Bromelain-Derived Peptide and RBD Variants

The HADDOCK scoring function combines various energies and buried surface area to arrive at a single numerical score. The HADDOCK score is defined as: 1.0 E_vdw_ + 0.2 E_elec_ + 1.0 E_desol_ + 0.1 E_AIR_, where E_vdw_ is an intermolecular van der Waals energy (kcal/mol), E_elec_ is an intermolecular electrostatic energy (kcal/mol), E_desol_ is a desolvation energy (kcal/mol), and E_AIR_ is a restraint violation energy (kcal/mol). The scores of the complex predicted using the HADDOCK2.2 web server are listed in Table 2. The HADDOCK score determined for each interaction of the peptide bromelain and RBD after docking can be described as follows: WT (−69.3 ± 3.2), SA (−78.6 ± 0.7), BR (−72.7 ± 3.3), UK (−70.7 ± 5.3), US (−71.0 ± 2.2), SG (−70.3 ± 1.5), SN (−70.5 ± 2.3), SC (−75.6 ± 0.4), IN (−68.6 ± 2.3), and NG (−82.1 ± 6.0). Since lower HADDOCK scores show a higher affinity between the peptide and protein, the interaction formed will be stronger and more stable [62]. Hence, the interaction between bromelain-derived peptide and the NG variant shows the highest affinity, followed by the SA, SC, BR, and US variants.

Binding affinity is used to assess and rank the strength of the interactions formed, which is also calculated by the equilibrium dissociation constant. Thus, the lower the K_D_ value, the higher the affinity [63]. The lowest K_D_ value was obtained from the interaction of the bromelain-derived peptide with the NG variant (9.7 × 10^−8^ M), followed by the US (1.8 × 10^−7^ M), UK (2.4 × 10^−7^ M), SC (2.7 × 10^−7^ M), WT (3.3 × 10^−7^ M), SG (3.5 × 10^−7^ M), SN (4.7 × 10^−7^ M), IN (5.1 × 10^−7^ M), and SA (6.6 × 10^−7^ M) variants. The BFE values calculated using the MM/GBSA method indicate that the bromelain peptide-RBD NG complex has the lowest value (−46.87 kJ/mol), followed by the SA variant (−42.74 kJ/mol), WT (−42.69 kJ/mol), BR (−38.91 kJ/mol), and CA (−37.99 kJ/mol). This suggests that the interaction between bromelain-derived peptide and the NG variant is more stable.

Z-scores represent the number of standard deviations from the mean for each cluster, with higher negative scores indicating better interactions. The Z-scores reveal that the NG variant shows the strongest interaction with bromelain-derived peptide (−2.4), followed by the SA (−2.2), SC (−2.0), BR (−1.8), SN (−1.7), US (−1.6), WT (−1.6), UK (−1.4), SG (−1.4), and IN variants (−1.2). The prodigy binding scores (ΔG) show that the NG variant also has the highest binding affinity with −9.9 kcal/mol, followed by the US (−9.6 kcal/mol), UK (−9.4 kcal/mol), SC (−9.3 kcal/mol), SG (−9.2 kcal/mol), WT (−9.2 kcal/mol), SN (−9.0 kcal/mol), IN (−8.9 kcal/mol), SA (−8.8 kcal/mol), and BR (−8.6 kcal/mol) variants. Thus, based on the results of the binding affinity calculation, bromelain-derived peptide has the highest affinity for the NG variant.

**Figure 3:**
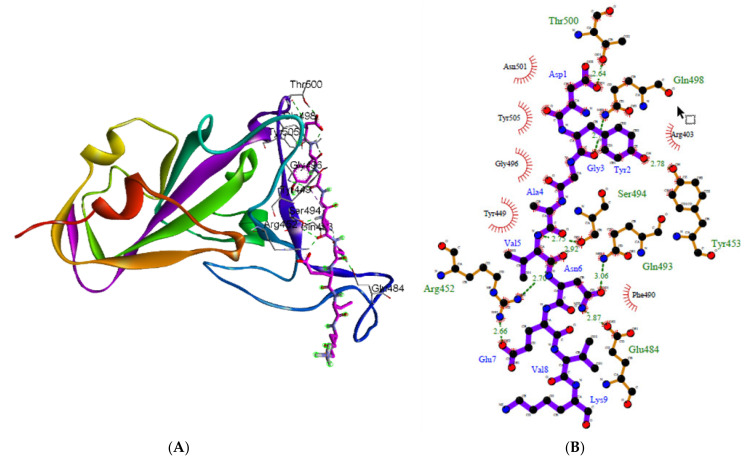
Sketch diagram depicting the 3D (**A**) and 2D (**B**) interactions between bromelain-derived peptide and RBD WT from LigPlot+.

**Figure 4:**
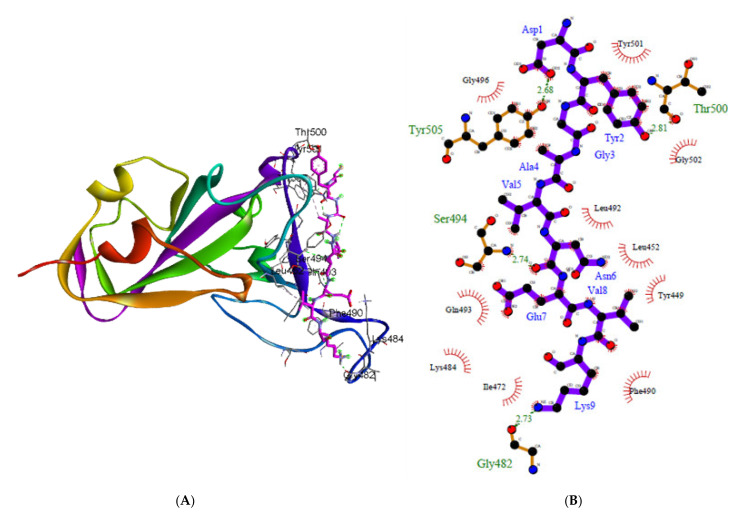
Sketch diagram depicting the 3D (**A**) and 2D (**B**) interaction between bromelain-derived peptide and RBD BR by LigPlot+. RBD BR contains the mutations K417T, E484, and N501Y.

The 2D visualization of the interaction generated using LigPlot+ is presented in Figure 3 and Figure 4, and Supplementary Materials (Figures S1–S8). Hydrogen bonds (H-bonds) are shown as green dotted lines. H-bonds play a critical role in drug-receptor interactions and in the structural integrity of a large number of biological molecules [64]. In addition to the van der Waals interaction in a complex, intermolecular hydrogen bonds contribute to the scoring function used to assess docking effectiveness [65]. Rathod et al. [8] discovered that the majority of studied peptides have a higher affinity for ACE2 than for the RBD residue binding motif. However, another study revealed that α-helical peptides extracted from the protease domain (PD) of ACE2 bind very specifically to SARS-CoV-2 and are stable, which implies that they can block the virus [9]. As a result, it is proposed that short peptides can be administered directly via inhalation to critical organs for SARS-CoV-2 infection, which offers an appealing alternative to traditional drug development [10].

The interaction studies were primarily concerned with the efficient binding of bromelain-derived peptide with the receptor-binding motifs (RBMs) of RBDs. The results show that bromelain-derived peptide formed H-bonds with the active site residues of RBM, which suggests a good propensity for efficient binding to RBDs and the ability to inhibit virus attachment to the hACE2 receptor. The LigPlot+ analysis of bromelain-derived peptide and the RBD reveals the interaction of hydrogen bonds with residues on the active side of RBD at Gln493 [O...N-H], Gln498 [O...N-H], and Thr500 [O...H-O] on the WT with distances of 3.06 and 2.64 Å, respectively; at Thr500 [O...H-O] on the BR variant with a distance of 2.81 Å; at Gly502 [O...N-H] on the SA variant with a distance of 2.77 Å; at Gly496 [N-H...O] on the UK variant with a distance of 3.22 Å; at Gln493 [N-H...O] and Thr500 [O...H-O] on the US variant with distances of 2.84 and 2.64 Å, respectively; at Gly496 [N-H...O] on the SG (NY1) variant with a distance of 3.31 Å; at Lys417 [O...H-N], Gln493 [O...H-N], and Gly496 [O...H-N] on the SN (NY2) variant with distances of 2.68, 2.63, 3.01, and 2.78 Å, respectively; at Tyr449 [O...H-O] and Gln493 [N-H...O] on the SC var-iant with distances of 3.01 and 3.05 Å, respectively; at Gln493 [O...H-N], Gly496 [O...H-N], and Gly502 [O...H-N] on the NG variant with distances of 2.84, 2.85, and 2.73 Å, respectively; and at Lys417 [O…H-N] and Gly496 [O...H-N] on the IN variant with distances of 2.74 and 3.00 Å, respectively.

According to the results of amino acid interactions, the bromelain-derived peptide was able to interact with the receptor-binding motif (RBM) of RBD by blocking unique residues designated as important in the binding of the human angiotensin-converting enzyme 2 (ACE2) cell receptor. The residues that are crucial for SARS-CoV-2 RBD binding to hACE2 are Gly446, Tyr449, Leu455, Phe486, Tyr491, Gln493, Gly496, Gln498, Thr500, Asn501, and Gly502 [66,67,68,69], as well as a salt bridge contributed by Lys417 [69]. The residues Phe486, Gln493, and Asn501 are the most important residues in the RBD identified by the hACE2 receptor on infected human cells because they facilitate RBD-hACE2 interaction [17,70]. Furthermore, it was reported that the amino acid substitutions S477G and S477N enhance the binding of the SARS-CoV-2 spike to the hACE2 receptor [71].

Table 3 summarizes the interaction between the amino acid residues of RBDs and bromelain-derived peptide, as determined by PDBsum analysis. With regards to the 3 most critical residues, i.e., Phe486, Gln493, and Asn501, the bromelain-derived peptide interacts with RBD WT via an H-bond on Gln493 and a hydrophobic interaction on Asn501. In particular, the BR and SA variants showed hydrophobic interactions with Gln493, while the SG and IN variants only showed hydrophobic interactions with Phe486. On the other hand, the US and NG variants contain H-bonds with Gln493 and hydrophobic interactions with Asn501. There is an H-bond on Gln493 and a hydrophobic interaction on Phe486 in the SN and SC variants. For Phe486 and Gln493, the UK variant demonstrates only hydrophobic interactions. Based on these findings, 8 of the 10 examined variants showed interactions with Gln493, including 5 variants with H-bonds and 3 variants with hydrophobic interactions. While 5 of the 10 variants interacted with Phe486 via hydrophobic interactions, only 2 variants interacted with Asn501 via hydrophobic interactions. Since hydrophobic interactions are entropy-driven at room temperature, they play a significant role in the docked complex binding affinity in a given solvent system [72]. The lower frequency of H-bonds is due to the binding pocket being more hydrophobic [73]. The peptide and RBD of the spike protein have a high number of H-bonds and hydrophobic interactions, thus indicating a strong interaction [21]. When compared to bromelain, the binding position of bromelain-derived peptide is generally similar, namely at the hACE2-RBD binding site. This is demonstrated by the similarity of the interacting amino acids between RBD WT and variants and these two molecules, particularly the critical residues Phe486, Gln493, and Asn501 [27].

Several studies have identified peptides as potential RBD inhibitors. The peptide ALPEEVIQHTFNLKSQ (P13) from *B. licheniformis* KN1G found in fermented kinema was reported to interact with residues Gln493 and Asn501 [74]. Furthermore, Souza et al. [21] reported that the synthetic peptides Mo-CBP3-PepII and PepGAT caused conformational changes in the structure of the SARS-CoV-2 spike glycoprotein, thereby decreasing its interaction with the hACE2 receptor. Another finding by Rathod et al. [8] showed that at RBD interfaces, the peptide AVP0671 significantly reduces binding affinity and changes the orientation of the RBD and ACE2 binding.

Proteins from mealworms, silkworm cocoons, and housefly larvae produce peptides when digested in the digestive tract. An in silico study reported that these peptides can bind to the RBD at key residues [75]. The peptide VEDKGMMHQQRMMEKAMNIPRMCGTMQRKCRMS, derived from quinoa seed protein, was shown to form hydrophobic interactions with the key binding residues Leu455, Phe456, Phe486, and Gln493 on the RBD [76]. However, not all peptides can bind to key RBD residues. The chimeric peptides cnCoVP-2, cnCoVP-5, and cnCoVP-6 have been shown to interact with RBD, but not with the key residue (Phe486) [17]. Importantly, despite the substitution of neutral and polar asparagine for neutral and polar tyrosine in residue 501 in this study, the bromelain-derived peptide could still recognize and bind to it. As a result, the bromelain-derived peptide could potentially prevent the interaction between hACE2 and RBD that undergoes mutation at residue 501.

### 3.6. Prediction of the Position of Bromelain-Derived Peptide Inhibition between the RBD and hACE2

**Figure 5:**
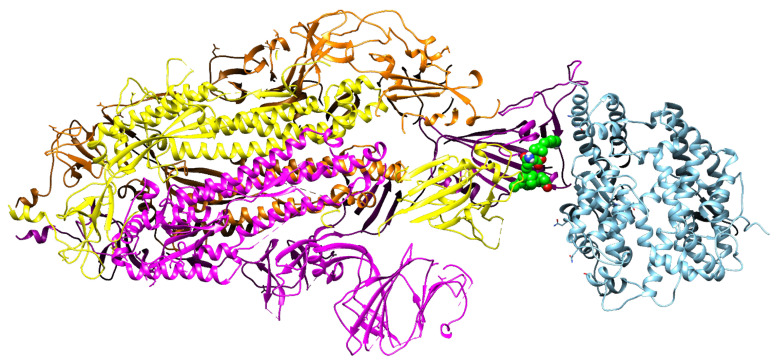
Schematic representation demonstrating bromelain peptide (green) inhibiting RBD (yellow) adhesion to hACE2 (light blue).

As illustrated in Figure 5, the type of bromelain-derived peptide inhibition at the binding site of the RBD and hACE2 is competitive. Several studies have been conducted using competitive inhibitors against the main protease from SARS-CoV-2 [77]. This competitive inhibition of bromelain-derived peptide can prevent the adhesion of hACE2 to the RBD since the positions of key amino acids in the RBD have been filled by the bromelain-derived peptide. This was confirmed by He et al. [78], who stated that a competitive inhibitor that binds to the active site of an enzyme can inhibit the activity of the enzyme by competing with the substrate, which in this case is hACE2. Due to the similarity of their binding interfaces to the hACE2 receptor’s S1 binding site (PDB ID: 6M0J), the antimicrobial peptide dermaseptin and its analog have been shown to act as competitive inhibitors for the hACE2 receptor [79].

### 3.7. Molecular Dynamics Simulations Study

The beneficial uses of MD simulation include developing a greater understanding of protein-ligand interactions, determining the spatial orientation of active sites, determining the motion of active site residues, and analyzing protein conformational dynamics. MD simulations with all atoms were run for 100 ns on the peptide-protein complexes of bromelain-derived peptide and the RBD. To understand the deviation of Cα atoms from the backbone as well as the fluctuations in amino acid contact during simulation, RMSD and RMSF analyses were conducted.

Currently, MD simulations are widely used in drug and vaccine design studies [80,81]. The notion that in silico techniques will aid in the treatment of diseases in desperate need of a cure, such as COVID-19, is becoming increasingly important. In particular, techniques such as molecular docking and MD simulation can save time and costs in demanding drug development efforts [82]. MD simulations are frequently used to investigate the stability of protein-ligand, protein-peptide, protein-protein, and protein-DNA/RNA complexes [83]. By MD simulation, the stability of the interactions between SARS-CoV-2 S1b unit (RBD) variants and the bromelain-derived peptide was investigated by employing the HADDOCK server. Amber99sb-ildn was chosen since it is suitable for force field protein-peptide and protein-protein simulations. A total of 10 protein-peptide complexes obtained by the molecular docking method were subjected to a pre-simulation for 20 ns. In this pre-simulation, WT and RBD variants with high stability were investigated over a longer time via a 100 ns simulation.

**Figure 6:**
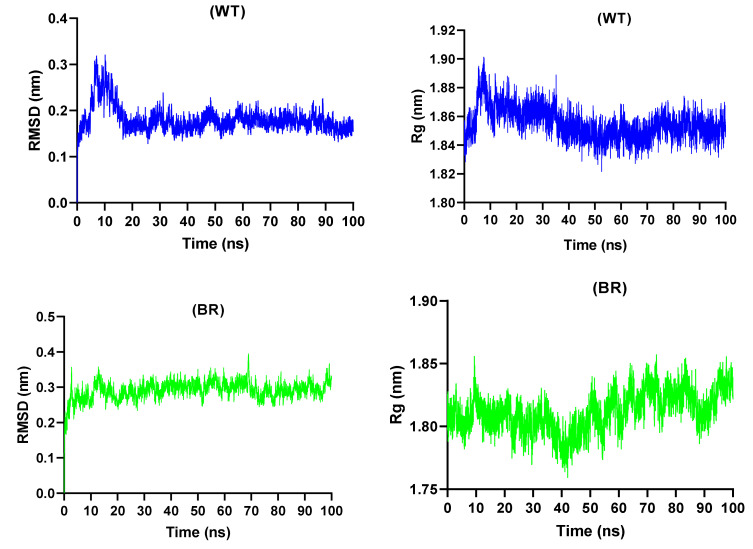

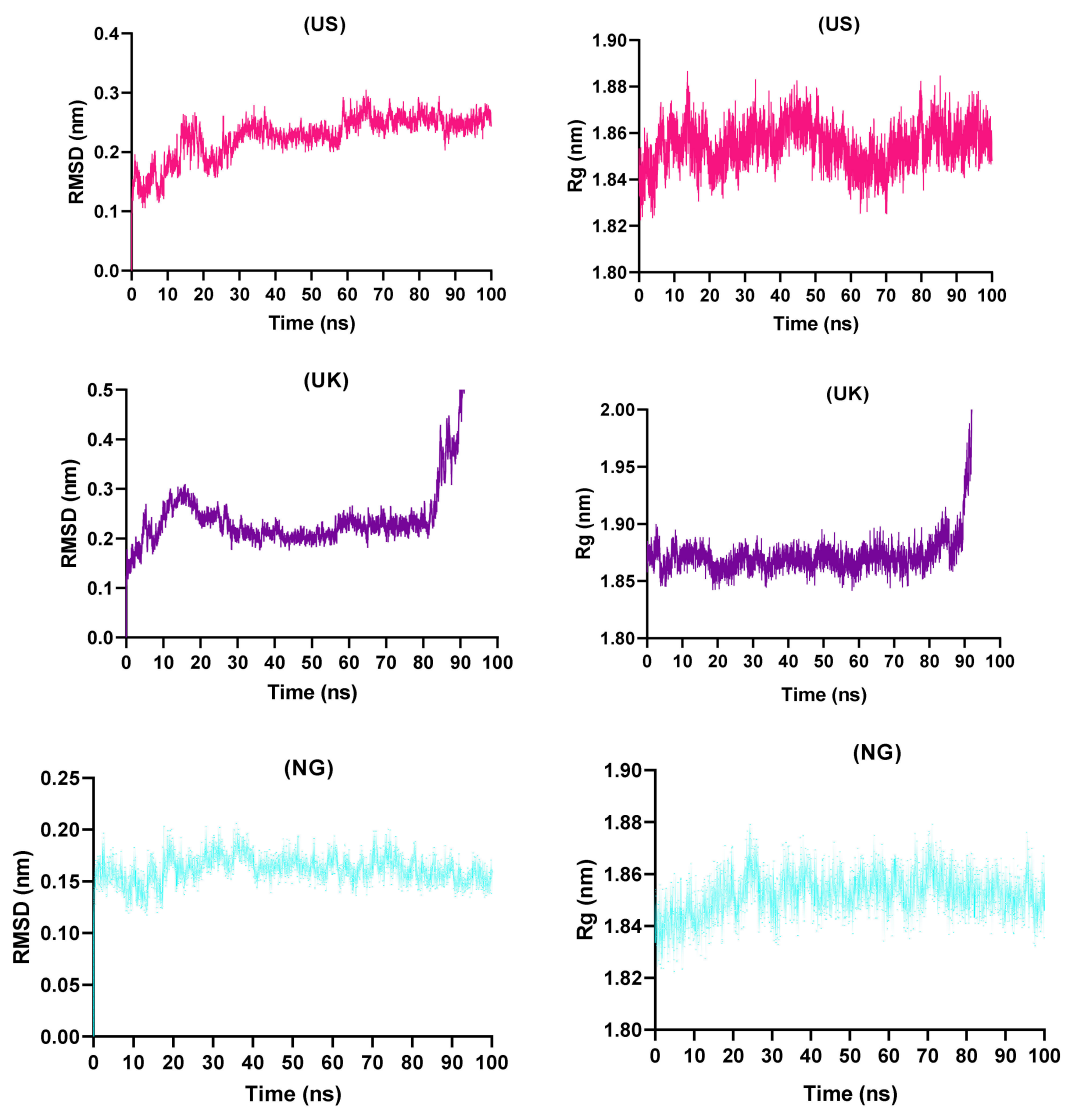
The root-mean-square deviation (RMSD) and radius of gyration (Rg) values obtained during molecular dynamics simulations of bromelain peptide with the RBD wild type (WT) and BR, US, UK, and NG variants.

The stability of the complex of bromelain peptide and RBDs was demonstrated by RMSD, Rg, and RMSF trajectory analysis. RMSD measurement is the main parameter expressing the deviation and shift in protein structure. The RMSD value of the complexes was calculated according to the backbone atoms. Rg calculations are another important parameter that provides information about the compactness of a protein. The lower and more constant the RMSD and Rg values are, the more stable the complex structure is. As shown in Figure 6, the protein-peptide complexes remained stable after a certain period of time. The RMSD value of the BR/RBD-peptide complex was approximately 0.3 nm, and the Rg value remained constant between 1.76 and 1.86 nm. The RMSD value of the US/RBD-peptide complex slowly increased up to 60 ns and approached 0.3 nm, remaining stable thereafter. The Rg value varied between 1.82 nm and 1.88 nm throughout the simulation. In the WT/RBD-peptide complex, it rose slightly above 0.3 ns during the first 10 ns and then remained stable at approximately 0.2 nm after 20 ns. Similar to the RMSD chart, the Rg chart shows fluctuation and stability. The highest Rg value was measured at 1.90 nm. The RMSD value of the UK/RBD-peptide complex increased to 0.3 nm in the first 20 ns and then remained constant at approximately 0.2 nm until 80 ns, after which the protein-peptide interaction was lost. Rg remained constant between 1.85 and 1.90 nm until 80 ns and increased greatly thereafter. Finally, the NG/RBD-peptide assembly remained stable below 0.2 nm throughout the simulation, with the Rg value also measuring between 1.85 and 1.87 nm.

**Figure 7:**
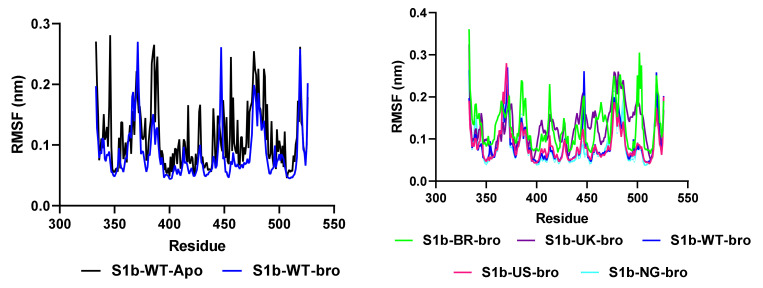
The root-mean-square fluctuation (RMSF) analysis showing the interaction and conformational changes between bromelain peptide and the spike protein RBD unit wild type apo and holo forms (**left**) as well as RBD variants and peptide complexes (**right**).

RMSF is another important MD trajectory analysis parameter showing fluctuations and conformational changes in protein structure. In the residue-based RMSF analysis, the numerical values of amino acids with high mobility are high, while the RMSF values of residues with lower mobility are low. RMSF calculations were made based on protein C-α atoms. To evaluate the status of the complex formed by bromelain peptide with RBD WT and RBD variants, the peptide-free WT/RBD (also known as the apo form) was simulated under the same conditions. As seen in Figure 7, the apo form has more mobility when compared to the amino acid holo forms (numbered 346, 382–386, 389, 454–456, and 517–519). Since the variants and WT/RBD-peptide complexes became more stable than the apo form, the peptide-protein complexes can be said to remain stable. The RBD flexibility of the BR/RBD (0.30 nm) and UK/RBD (0.21 nm) variants, which contain the N501Y mutation, was higher than that of WT/RBD (0.09 nm). This may explain why the infectiousness of the N501Y mutant is higher. The E484K mutation also increases the mobility of the RBD. In particular, the flexibility of BR/RBD (0.24 nm) and NG/RBD (0.21 nm) variants containing E484K increased at amino acid 484 compared to WT/RBD (0.13 nm).

### 3.8. MM-PBSA Binding-Free Energy

Although BFE calculations were performed in the preceding section, we consider calculations based on MM-PBSA in this section. The MM-PBSA was calculated to measure the in-depth atomic-level interaction energy of bromelain-derived peptide-RBD complexes (Table 4). This is due to the lower accuracy of the BFE value derived from docking results [84], which is only used as an initial prediction. It employs a scoring function to rank the various possible poses of a ligand in a binding pocket, which is focused on determining the binding affinity. The BR and NG variants with the most stable RMSD plots and the bromelain-derived peptide produced the lowest average binding energy to the peptide (−287.356, and −255.801 kJ/mol, respectively). Similarly, according to the RMSD plot, the UK variant, with the lowest stability and disrupted protein-peptide complex after 80 ns, gave an average free energy value of −89.129 kJ/mol. The WT and US variants gave values of −173.243 and −150.460 kJ/mol, respectively. According to these MM-PBSA results, the protein-peptide connection continues for a certain period of time. This may indicate that the bromelain peptide remains stable at the possible binding site to the RBD.

## 4. Conclusions

In this study, we evaluated bromelain-derived peptide as a potential drug to target inhibition of the interaction between the RBD and the hACE2 receptor. The results of the MD simulation validated our decision to propose the bromelain-derived peptide as an inhibitor against the RBD WT and its variants. Throughout the simulation, the peptide-RBD complexes remained stable. This result indicates that bromelain-derived peptide could potentially be used as a drug to prevent a prolonged COVID-19 pandemic by inhibiting viral fusion and entry into cells. However, further in vitro and in vivo testing will be required to validate the efficacy and safety of the bromelain-derived peptide as a SARS-CoV-2 inhibitor.

## Figures and Tables

**Table 1:** Mutation sites in the RBD.

Variants		PANGO Lineage	Greek Alphabet	Mutation Sites					
WT	Wild Type								
SA	South Africa	B.1.351	Beta	K417N				E484K	N501Y
BR	Brazil	P.1	Gamma	K417T				E484K	N501Y
UK	United Kingdom	B.1.1.7	Alpha						N501Y
CA	California	B.1.429	Epsilon			L452R			
SG	New York	B.1.526	Iota				S477G		
SN	New York	B.1.526	Iota				S477N		
SC	Indian	B.1.617.2	Delta			L452R		E484Q	
NG	Nigeria	B.1.525	Eta					E484K	

**Table 2:** The HADDOCK docking predictions for all bromelain-derived peptide with RBD variants complexes, along with additional parameters such as cluster size, Van der Waals energy, electrostatic energy, and Z-score. The KD and MM/GBSA free binding energy (kcal/mol) are also included in the table.

Parameters	WT	SA	BR	UK	CA	SG	SN	SC	IN	NG
HADDOCK Score (a.u.)	−69.3 ± 3.2	−78.6 ± 0.7	−72.7 ± 3.3	−70.7 ± 5.3	−71.0 ± 2.2	−70.3 ± 1.5	−70.5 ± 2.3	−75.6 ± 0.4	−68.6 ± 2.3	−82.1 ± 6.0
MM/GBSA (kcal/mol)	−42.69	−42.74	−38.91	−19.03	−37.99	−26.66	26.84	−29.27	−29.54	−46.87
Cluster Size	9	16	15	15	13	60	66	66	7	8
RMSD (Å)	2.2 ± 0.1	0.3 ± 0.0	0.4 ± 0.3	3.0 ± 0.0	1.9 ± 0.1	2.2 ± 0.1	0.9 ± 0.5	0.8 ± 0.5	4.1 ± 0.0	0.2 ± 0.1
Intermolecular Van der Waals Energy (kcal/mol)	−36.7 ± 2.6	−32.2 ± 2.2	−33.0 ± 0.3	−37.3 ± 3.1	−45.0 ± 6.5	−40.3 ± 2.4	−36.2 ± 2.3	−39.6 ± 1.8	−36.7 ± 2.0	−37.4 ± 1.3
Intermolecular electrostatic Energy (kcal/mol)	−156.9 ± 5.0	−205.6 ± 12.8	−179.2 ± 38.8	−117.2 ± 22.2	−122.9 ± 40.7	−86.8 ± 8.0	−134.7 ± 35.2	−132.8 ± 22.1	−128.8 ± 22.9	−220.1 ± 10.1
Desolvation Energy (kcal/mol)	−4.2 ± 1.3	−9.5 ± 2.0	−10.0 ± 5.1	−15.3 ± 4.5	−5.6 ± 2.8	−15.8 ± 1.8	−10.8 ± 4.2	−11.9 ± 2.5	−11.5 ± 2.9	−3.0 ± 2.0
Restraint Violation Energy (kcal/mol)	29.4 ± 15.1	42.3 ± 17.2	60.8 ± 12.3	53.4 ± 31.6	41.9 ± 2.1	32.8 ± 32.2	34.1 ± 19.5	24.8 ± 18.9	53.7 ± 19.3	22.5 ± 11.3
Buried Surface Area (Å)	1079.4 ± 65.4	1122.3 ± 27.5	1083.8 ± 28.1	1090.8 ± 51.4	1107.7 ± 35.2	1029.4 ± 22.3	1022.8 ± 58.6	1102.2 ± 52.0	1098.0 ± 47.5	1094.2 ± 30.7
Z-Score	−1.6	−2.2	−1.8	−1.4	−1.6	−1.4	−1.7	−2.0	−1.2	−2.4
Prodigy ΔG (kcal/mol)	−9.2	−8.8	−8.6	−9.4	−9.6	−9.2	−9.0	−9.3	−8.9	−9.9
K_D_ (M) at 37.0 °C	3.3 × 10^−7^	6.6 × 10^−7^	9.0 × 10^−7^	2.4 × 10^−7^	1.8 × 10^−7^	3.5 × 10^−7^	4.7 × 10^−7^	2.7 × 10^−7^	5.1 × 10^−7^	9 × 10^−8^

**Table 3:** List of interacting amino acids between RBDs and bromelain-derived peptide. The position of the interacting residues in pocket and mutation sites are indicated in italics, while the key amino acid residues that play a role in binding RBD to hACE2 are marked in bold.

Variants	H-Bonds		Non-Bonded Contacts	
	RBD	Bromelain-Derived Peptide	RBD	Bromelain-Derived Peptide
WT	*Arg452* (2)	Glu7, Val5	Arg403	Tyr2
	Tyr453 (2)	Tyr2 (2)	**Tyr449** (6)	Gly3 (2); Ala4 (2) Val5 (2);
	*Glu484*, **Gln493**	Asn6 (2)	*Arg452* (9)	Val5 (4); Glu7 (5)
	Ser494 (2)	Val5 (2)	Tyr453 (4)	Tyr2 (4)
	**Gln498**	Tyr2	*Glu484* (5)	Asn6 (5)
	**Thr500**	Asp1	Phe490	Asn6
			**Gln493** (4)	Ala4 (2); Asn6 (2)
			Ser494 (8)	Gly3; Ala4 (2); Val5 (5)
			**Gly496** (5)	Tyr2; Gly3 (4)
			**Gln498** (9)	Asp1 (4); Tyr2 (5);
			**Thr500** (7)	Asp1 (7)
			***Asn501*** (10)	Asp1 (10)
			**Gly502**	Asp1
			Tyr505 (4)	Asp1 (3); Tyr2
BR	Gly482	Lys2	**Tyr449** (3)	Asn6 (3)
	Ser494	Asn6	*Leu452* (4)	Val8 (4)
	**Thr500**	Tyr2	Ile472 (2); Gly482	Lys9 (3)
	Tyr505	Asp1	*Lys484* (5)	Glu7 (5)
			Phe490 (4)	Val8 (3); Lys9
			Leu492 (3)	Glu7 (2); Val8
			**Gln493** (7)	Val5; Asn6 (5); Glu7
			Ser494 (5)	Ala4; Asn6 (4)
			**Gly496** (2)	Ala4 (2)
			**Thr500** (2)	Tyr2 (2)
			***Tyr501*** (12)	Tyr2 (11); Ala4
			**Gly502**	Tyr2 (2)
			Tyr505 (8)	Asp1 (5); Tyr2 (3)
SA	Gly482	Lys9	**Tyr449** (4)	Asn6 (4)
	*Lys484*	Glu7	*Leu452*	Val8
	Ser494	Asn6	Tyr453	Val5
	**Gly502**	Asp1	Thr470; Ile472 (2); Gly482	Lys9 (4)
			*Lys484* (4)	Glu7 (4)
			Phe490 (6)	Val8 (3); Lys9 (3)
			Leu492	Glu7
			**Gln493** (9)	Val5 (2); Asn6 (4); Glu7 (3)
			Ser494 (7)	Val5 (2); Asn6 (5)
			**Gly496** (2)	Ala4 (2)
			**Thr500** (4)	Tyr2 (4)
			***Tyr501*** (20)	Asp1 (2); Tyr2 (9); Gly3 (6); Ala4 (3)
			**Gly502** (6)	Asp1 (6)
			Tyr505 (8)	Asp1 (6); Tyr2 (2)
UK	Asn487; Tyr489	Glu7 (2)	Arg403 (3); Glu406 (4); ***Lys417*** (5); Ile418 (2); Tyr453 (9)	Tyr2 (23)
	**Gly496**; ***Tyr501***	Asp1 (2)	Tyr453	Gly3
			**Leu455** (3)	Gly3; Ala4 (2)
			Phe456 (6)	Val5 (3); Asn6 (3)
			Ala475 (2)	Glu7; Val8
			Gly485	Glu7
			**Phe486** (6)	Glu7 (3); Lys9 (3)
			Asn487 (14)	Glu7 (10); Val8 (4)
			Tyr489 (8)	Glu7 (8)
			**Gln493**	Gly3
			Tyr495 (3)	Asp1; Tyr2 (2)
			**Gly496** (4); ***Tyr501*** (5); Tyr505 (4)	Asp1 (13)
CA (USA)	*Arg452*; **Gln493**	Asn6 (2)	**Tyr449** (8)	Gly3 (2); Ala4 (2); Val5 (4)
	Ser494	Val5	*Arg452* (2)	Asn6 (2)
	**Thr500**	Asp1	Tyr453 (3)	Tyr2 (2)
			Ile472; *Glu484* (7)	Val8 (8)
			Phe490 (9)	Asn6 (6); Val8 (3)
			Leu492 (3)	Asn6 (3)
			**Gln493** (8)	Ala4; Val5 (3); Asn6 (4)
			Ser494	Ala4; Val5 (3)
			Tyr495 (2)	Tyr2 (2)
			**Gly496** (9)	Tyr2 (4); Gly3 (5)
			**Gln498** (4)	Asp1 (2); Gly3 (2)
			**Thr500** (8)	Asp1 (8)
			***Asn501*** (13)	Asp1 (12); Tyr2
			Tyr505 (4)	Asp1 (3); Tyr2
SG (NY1)	Arg403	Asp1	Arg403 (4)	Asp1 (4)
	Tyr453	Tyr2	**Tyr449** (8)	Tyr2 (8)
	*Glu484*	Asn6	Tyr453	Tyr2
			*Glu484* (6)	Asn6 (5); Val8
			Gly485 (7)	Asn6 (2); Glu7 (3); Val8 (2)
			**Phe486** (13)	Glu7 (3); Val8 (7); Lys9 (3)
			Asn487; Cys488	Asn6 (2)
			Tyr489 (7)	Val5 (4); Asn6 (3)
			**Gln493** (12)	Tyr2 (3); Gly3 (5); Ala4 (4)
			Ser494 (4)	Tyr2 (4)
			**Gly496** (4)	Asp1 (2); Tyr2 (2)
			***Asn501***; Tyr505 (4)	Asp1 (5)
SN(NY2)	***Lys417*** (3)	Asp1 (2); Tyr2	Arg403 (10)	Asp1 (8); Tyr2 (2)
	**Gln493**	Ala4	***Lys417*** (7)	Asp1 (6); Tyr2
	**Gly496**	Tyr2	Tyr453 (8)	Tyr2 (6); Gly3 (2)
	Tyr505	Asp1	**Leu455** (4)	Tyr2; Gly3; Ala4 (2)
			Phe456	Val5
			*Glu484* (8)	Asn6 (2); Glu7 (2); Val8 (4)
			Gly485 (12)	Glu7 (10); Val8 (2)
			**Phe486** (2)	Glu7 (2)
			Tyr489 (7)	Val5 (3); Asn6 (4)
			**Gln493** (4)	Ala4 (4)
			Ser494	Tyr2
			Tyr495 (5)	Tyr2 (5)
			**Gly496** (3)	Tyr2 (3)
			Tyr505 (2)	Asp1 (2)
SC	Arg403	Asp1	Arg403 (5)	Asp1 (5)
	**Tyr449**	Tyr2	**Tyr449** (9), Tyr453	Tyr2 (10)
	*Glu484*	Asn6	*Glu484* (5)	Asn6 (5)
	Asn487	Glu7	Gly485 (8)	Asn6; Glu7 (4); Val8 (3)
	**Gln493**	Ala4	**Phe486** (12)	Glu7 (4); Val8 (5); Lys9 (3)
			Asn487 (2)	Glu7 (2)
			Cys488	Asn6
			Tyr489 (5)	Val5 (3); Asn6
			Asn493 (14)	Tyr2 (6); Gly3 (5); Ala4 (3)
			Ser494	Tyr2
			**Gly496** (9)	Asp1 (5); Tyr2 (4)
			***Asn501***; Tyr505 (3)	Asp1 (4)
IN	Gln409; ***Lys417***	Asp1 (2)	Arg403 (10)	Asp1 (2); Tyr2 (4)
	Asn487	Glu7	Glu406 (4)	Asp1 (2); Tyr2 (2)
	**Gly496**	Tyr2	Gln409	Asp1 (2)
			Gly416 (2)	Asp1 (2)
			***Lys417*** (10)	Asp1 (8); Tyr2 (2)
			Tyr453 (15)	Tyr2 (13); Gly3 (2)
			**Leu455** (4)	Tyr2; Gly3; Ala4 (2)
			Gly485 (6)	Glu7 (2); Val8 (4)
			**Phe486** (6)	Glu7 (3); Val8 (3)
			Asn487 (3)	Glu7 (3)
			Tyr489 (7)	Asn6 (3); Glu7 (4)
			**Gly496** (2)	Tyr2 (2)
NG	Gly482	Lys9	**Tyr449** (4)	Ala4 (2); Asn6 (2)
	*Lys484*; **Gln493**	Glu7 (2)	*Leu452* (3)	Val8 (3)
	Ser494 (2)	Asn6 (2)	Gly482 (3)	Lys9 (3)
	**Gly496**	Ala4	*Lys484* (5)	Glu7 (5)
	**Gly502**	Asp1	Phe490 (11)	Glu7; Val8 (3); Lys9 (7)
			Leu492 (2)	Glu7 (2)
			**Gln493** (8)	Val5; Asn6 (3); Glu7 (3)
			Ser494 (7)	Ala4; Val5; Asn6 (5)
			**Gly496** (7)	Gly3; Ala4 (6)
			**Gln498** (8)	Tyr2 (5); Gly3 (3)
			***Asn501*** (13)	Asp1; Tyr2 (9); Gly3 (3)
			**Gly502** (2)	Asp1
			Tyr505 (14)	Asp1 (12); Tyr2 (2)

**Table 4:** Results of the MM-PBSA interaction energy (kJ/mol) calculations for bromelain-derived peptide-RBD/WT, BR, UK, CA, and NG variant complexes.

Parameters (kJ/mol)	WT	BR	UK	CA	NG
Van der Waals Energy	−229.646 ± 21.620	−237.086 ± 22.932	−117.540 ± 4.519	−130.427 ± 22.184	−220.283 ± 21.339
Electrostatic Energy	−436.047 ± 66.496	−257.948 ± 44.899	−222.193 ± 82.117	−365.998 ± 162.924	−450.044 ± 49.243
Polar Solvation Energy	521.232 ± 69.214	230.554 ± 59.360	266.989 ± 108.001	363.554 ± 183.985	438.652 ± 57.986
SASA Energy	−28.782 ± 1.707	−22.875 ± 1.862	−16.385 ± 2.108	−17.589 ± 3.215	−24.126 ± 2.072
Binding Energy	−173.243 ± 33.428	−287.356 ± 32.004	−89.129 ± 48.966	−150.460 ± 38.762	−255.801 ± 29.792

## Data Availability

The authors will make the raw data supporting the conclusions of this manuscript available to any qualified researcher.

## Supplementary Materials

The following are available online, Figure S1: Sketch diagram depicting the 3D (A) and 2D (B) interactions between bromelain-derived peptide and RBD SA by LigPlot+. RBD SA contains the mutations K417N, E484 and N501Y. Figure S2: Sketch diagram depicting the 3D (A) and 2D (B) interactions between bromelain-derived peptide and RBD UK by LigPlot+. RBD UK contains the mutation N501Y. Figure S3: Sketch diagram depicting the 3D (A) and 2D (B) interactions between bromelain-derived peptide and RBD CA (US) by LigPlot+. RBD CA contains the mutation L452R. Figure S4. Sketch diagram depicting the 3D (A) and 2D (B) interactions between bromelain-derived peptide and RBD SG (NY1) by LigPlot+. RBD SG contains the mutation S477G. Figure S5. Sketch diagram depicting the 3D (A) and 2D (B) interactions between bromelain-derived peptide and RBD SN (NY2) by LigPlot+. RBD SN contains the mutation S477N. Figure S6. Sketch diagram depicting the 3D (A) and 2D (B) interactions between bromelain-derived peptide and RBD SC by LigPlot+. RBD SC contains the mutation N439K. Figure S7. Sketch diagram depicting the 3D (A) and 2D (B) interactions between bromelain-derived peptide and RBD NG by LigPlot+. RBD NG contains the mutation E484K. Figure S8. Sketch diagram depicting the 3D (A) and 2D (B) interactions between bromelain peptide and RBD IN by LigPlot+. RBD IN contains the mutations L452R and E484K.
