# Supplementary material for: Fruit Bromelain-Derived Peptide Potentially Restrains the Attachment of SARS-CoV-2 Variants to hACE2: A Pharmacoinformatics Approach
Source: Molecules. 2022 Jan 1;27(1):260. doi: 10.3390/molecules27010260 (PMC8746556; doi:10.3390/molecules27010260)
Supplement: Supplementary file 1 [file molecules-27-00260-s001.zip › molecules-1496184-supplementary.pdf]

Supplementary Materials

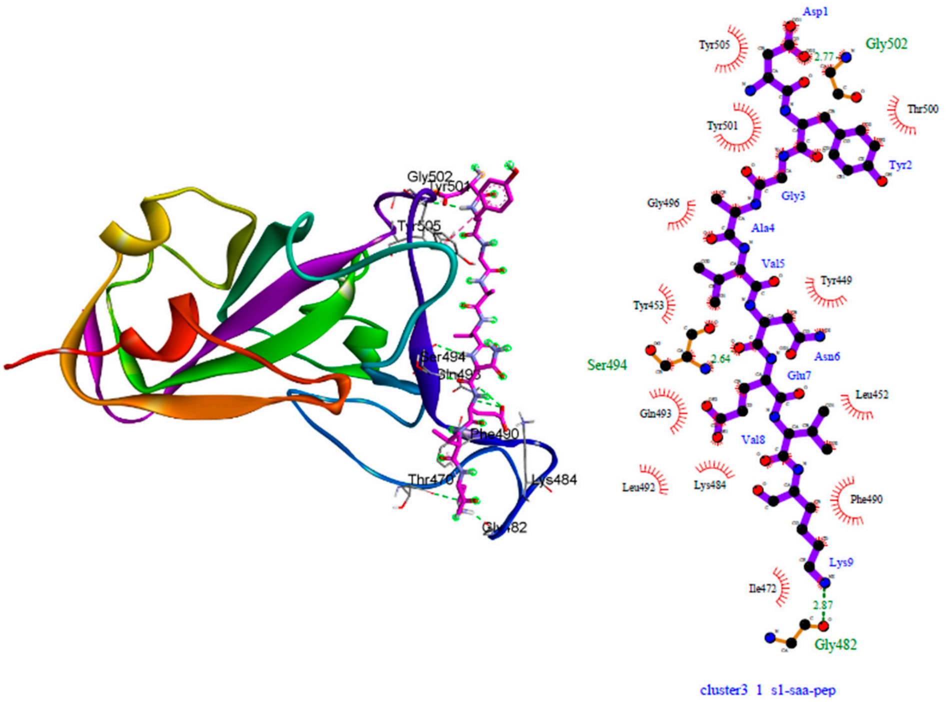

A **B**

Figure S1. Sketch diagram depicting the 3D (**A**) and 2D (**B**) interaction between bromelain-derived peptide and RBD SA by LigPlot+. RBD SA contains the mutations K417N, E484 and N501Y.

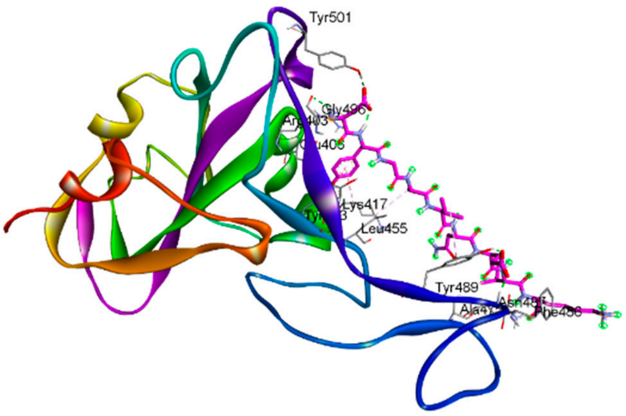

A

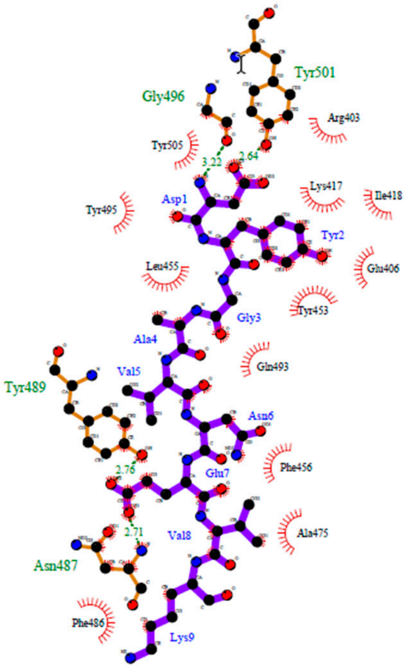

B

Figure S2. Sketch diagram depicting the 3D (**A**) and 2D (**B**) interaction between bromelain-derived peptide and RBD UK by LigPlot+. RBD UK contains the mutation N501Y.

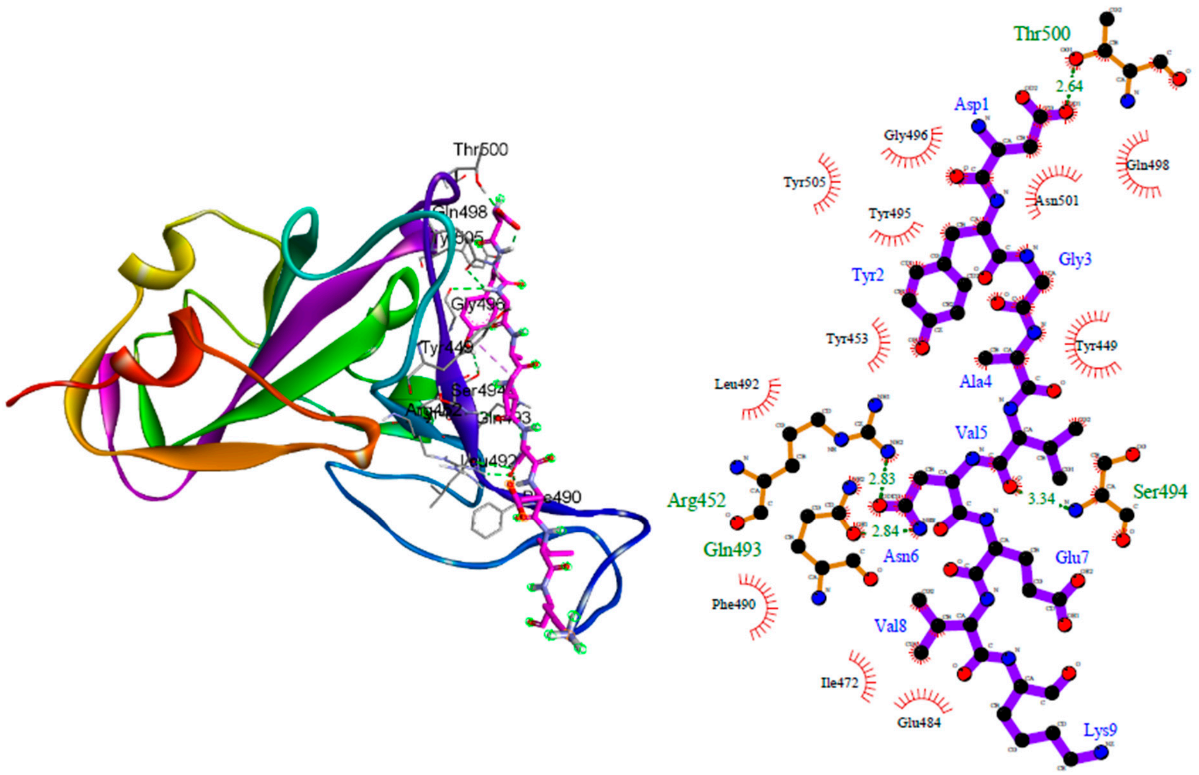

A **B**

Figure S3. Sketch diagram depicting the 3D (**A**) and 2D (**B**) interaction between bromelain-derived peptide and RBD CA (US) by LigPlot+. RBD CA contains the mutation L452R.

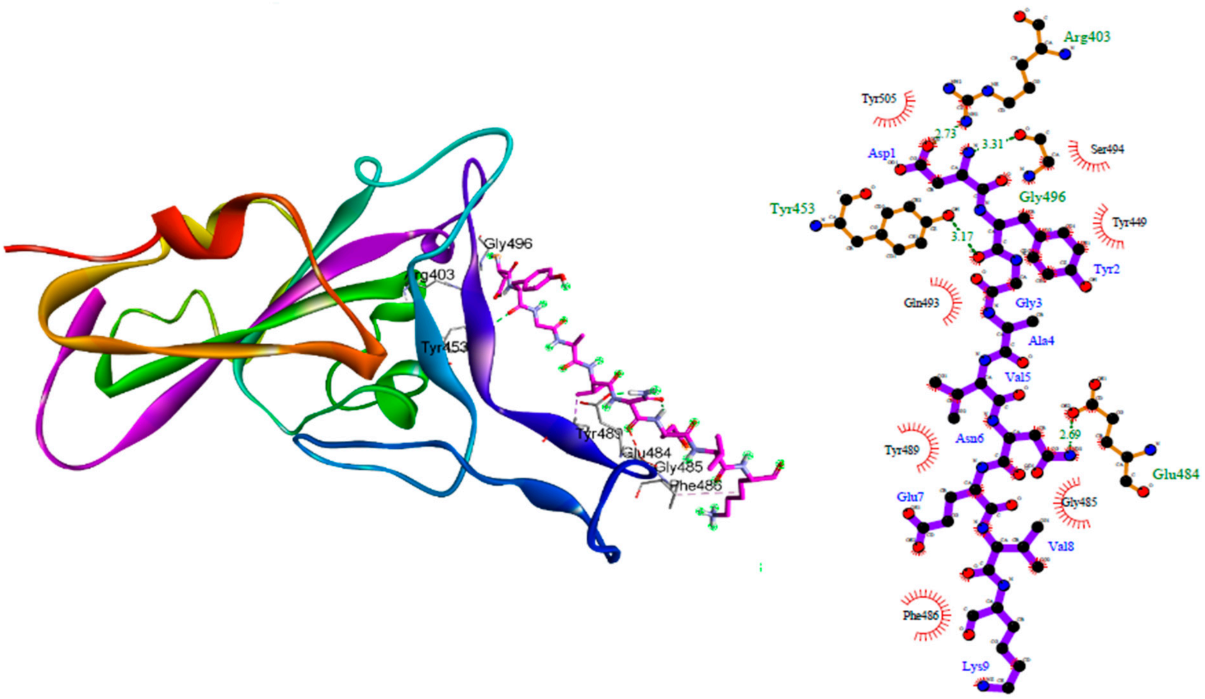

A **B**

Figure S4. Sketch diagram depicting the 3D (**A**) and 2D (**B**) interaction between bromelain-derived peptide and RBD SG (NY1) by LigPlot+. RBD SG contains the mutation S477G.

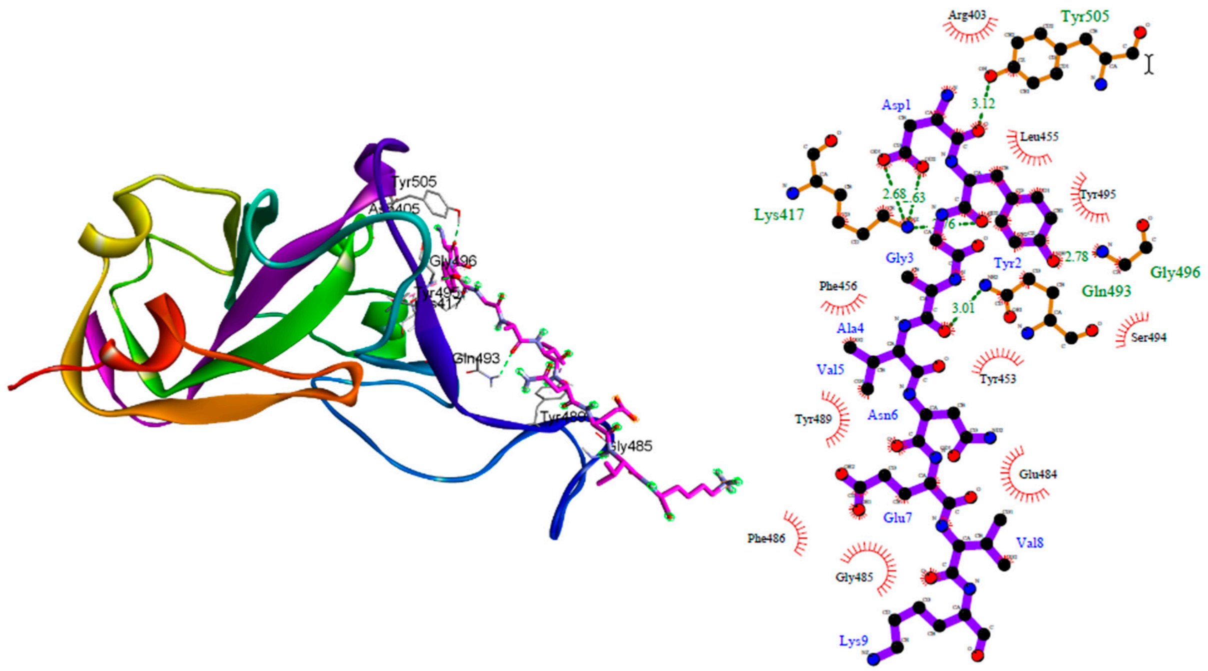

A **B**

Figure S5. Sketch diagram depicting the 3D (**A**) and 2D (**B**) interaction between bromelain-derived peptide and RBD SN (NY2) by LigPlot+. RBD SN contains the mutation S477N.

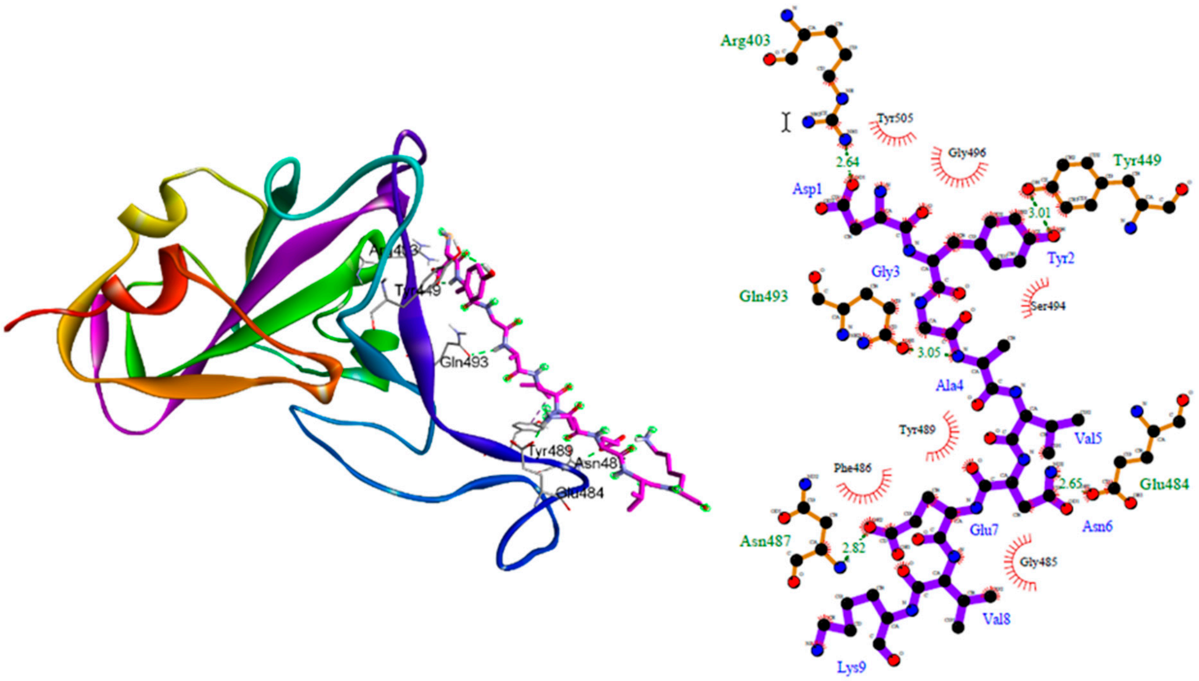

A **B**

Figure S6. Sketch diagram depicting the 3D (**A**) and 2D (**B**) interaction between bromelain-derived peptide and RBD SC by LigPlot+. RBD SC contains the mutation N439K.

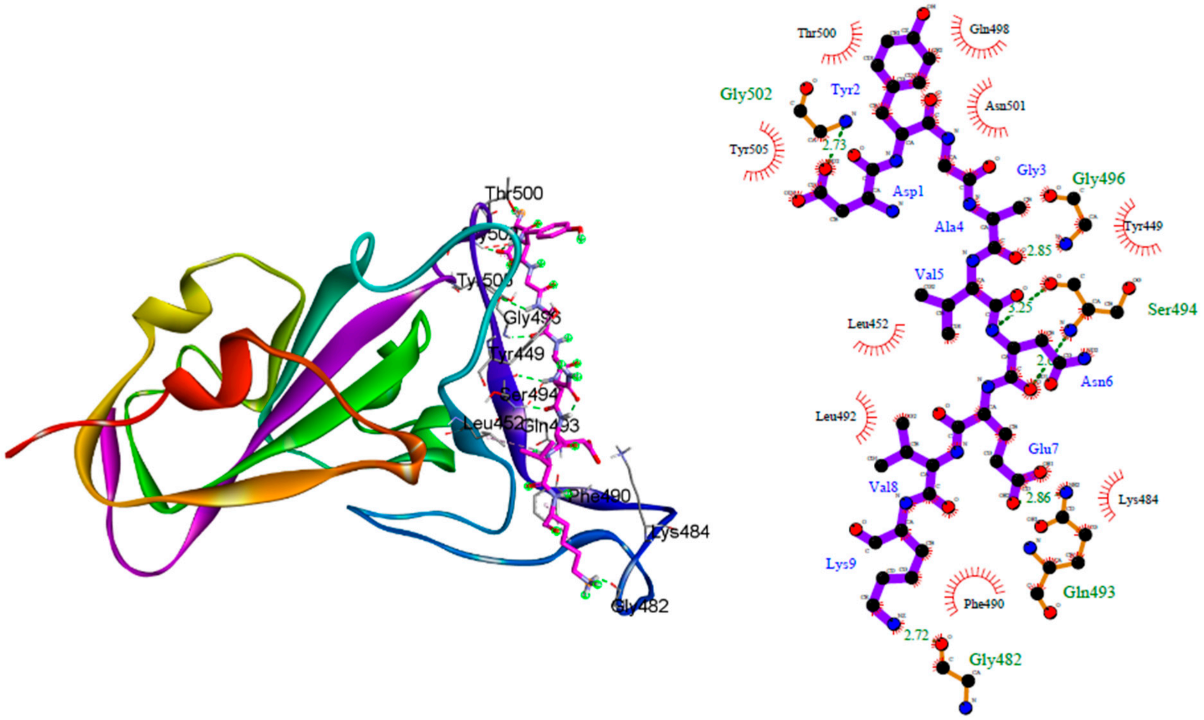

A **B**

Figure S7. Sketch diagram depicting the 3D (**A**) and 2D (**B**) interaction between bromelain-derived peptide and RBD NG by LigPlot+. RBD NG contains the mutation E484K.

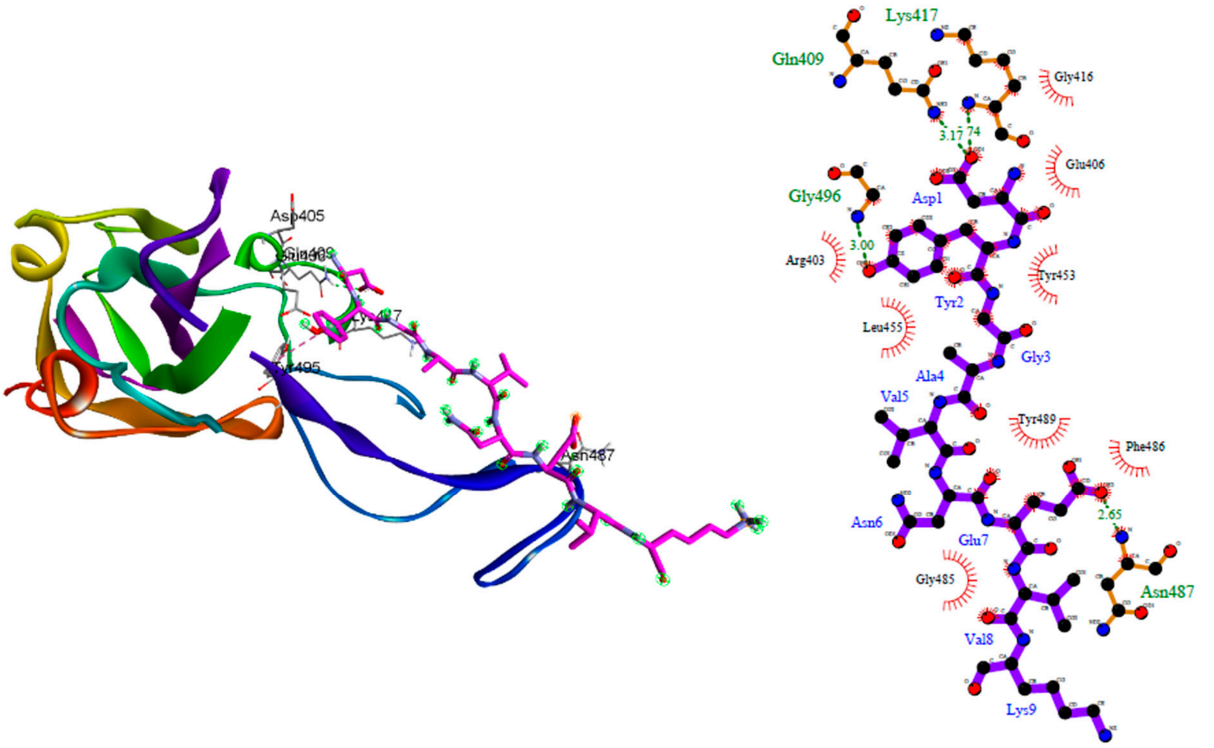

A **B**

Figure S8. Sketch diagram depicting the 3D (**A**) and 2D (**B**) interaction between bromelain peptide and RBD IN by LigPlot+. RBD IN contains the mutations L452R and E484K.
